# Identification of *Echinacea Purpurea* (L.) Moench Root LysM Lectin with Nephrotoxic Properties

**DOI:** 10.3390/toxins12020088

**Published:** 2020-01-28

**Authors:** Gabriele Balciunaite, Perttu-Juhani Haimi, Zoja Mikniene, Girius Savickas, Ona Ragazinskiene, Nomeda Juodziukyniene, Danas Baniulis, Dalia Pangonyte

**Affiliations:** 1 Institute of Pharmaceutical Technology, Faculty of Pharmacy, Academy of Medicine, Lithuanian University of Health Sciences, Sukileliu ave. 13, 50162 Kaunas, Lithuania; 2 Institute of Horticulture, Lithuanian Research Centre for Agriculture and Forestry, Kaunas str. 30, Babtai, 54333 Kaunas distr., Lithuania; 3 Clinical Research Laboratory, Large animal clinic, Faculty of Veterinary, Veterinary academy, Lithuanian University of Health Sciences, Tilzes str. 18, 47181 Kaunas, Lithuania; 4 Faculty of Medicine, Academy of Medicine, Lithuanian University of Health Sciences, A. Mickeviciaus str. 9, 44307 Kaunas, Lithuania; 5 Kaunas Botanical Garden, Vytautas Magnus University, Z. E. Zilibero str. 6, 46324 Kaunas, Lithuania; 6 Department of Veterinary Pathobiology, Faculty of Veterinary, Academy of Veterinary, Lithuanian University of Health Sciences, Tilzes str. 18, 47181 Kaunas, Lithuania; 7 Laboratory of Cardiac Pathology, Institute of Cardiology, Lithuanian University of Health Sciences, Sukileliu ave. 15, 50162 Kaunas, Lithuania

**Keywords:** *Echinacea purpurea* L. Moench, lectin, hemagglutinin, LysM, lectin purification, affinity chromatography, hemagglutinating activity, nephrotoxicity

## Abstract

*Echinacea purpurea* (L.) Moench (EP) is a well-studied plant used for health benefits. Even though there are a lot of data on EP secondary metabolites, its active proteins are not studied well enough. The aim of our experiment was to purify lectin fraction from EP roots and evaluate its biological activity in vitro as well as its effect on kidney morphology in vivo. An EP root glycoprotein fraction was purified by affinity chromatography, identified by LC-MS/MS, and used for biological activity tests in vitro and in vivo. Identified glycoproteins were homologous with the LysM domain containing lectins from the *Asteraceae* plants *Helianthus annuus* L., *Lactuca sativa* L., *Cynara cardunculus* L. A purified fraction was tested by hemagglutination and hemagglutination inhibition (by carbohydrate reactions) in vitro. We purified the hemagglutinating active ~40 kDa size lactose, D-mannose, and D-galactose specific glycoproteins with two peptidoglycan binding LysM (lysine motif) domains. Purified LysM lectin was tested in vivo. Eight-week old Balb/C male mice (n = 15) were treated with 5 μg of the purified lectin. Injections were repeated four times per week. At the fifth experimental week, animals were sedated with carbon dioxide, then euthanized by cervical dislocation and their kidney samples were collected. Morphological changes were evaluated in hematoxylin and eosin stained kidney samples. The purified LysM lectin induced a statistically significant (*p* < 0.05) kidney glomerular vacuolization and kidney tubular necrosis (*p* < 0.001).

## 1. Introduction

Lectins are reversible and nonimmune glycoproteins, found in all living organisms, which can bind specific carbohydrates. Plant lectins are accumulated in all parts of a plant: Leaves, flowers, stems, but especially in the storage tissues of roots and seeds [1]. These glycoproteins historically were classified as toxins, which could cause hemagglutination [2], gut morphology changes [3], liver damage [4], animal weight loss [5], or even death [6]. However, later, experimental data showed potential dose-dependent benefits, for instance: Antibacterial [7], antifungal [8], antiviral [9], insecticidal [10], cytotoxic [11], immunomodulatory [12], and antiproliferative [13] effects.

Lectins of *Asteraceae* plant family were previously purified and studied in *Helianthus tuberosus* L. tubers [14]. However, there is not enough available data on lectins from *Echinacea purpurea* (L.) Moench. Phytochemical studies of *Echinacea purpurea* (L.) Moench showed a broad composition of caffeic acid derivates, polyphenols, polysaccharides, proteoglycans, and lipophilic alkylamides in aerial parts and roots [15,16,17] with higher contents of chemical constituents in fresh herbal material [18]. It is widely used in therapeutic practice for immunostimulatory [19] and anti-inflammatory [20] effects.

Although purple coneflower extracts and juice have been used for a long time, there are ambiguous data on the biological activities of its glycoproteins. There is little information about the composition of lectins in raw material of the plant. In this study, we purified and identified lectin with two LysM (lysine motif) domains from *E. purpurea* roots. Due to the lack of data on the LysM lectin effect in vivo, we performed purified lectin testing in the animal model. These results demonstrated nephrotoxic effects of purified LysM lectin in vivo.

## 2. Results

### 2.1. Protein Extraction, Lectin Purification, and Evaluation of Hemagglutinating Activity

In order to evaluate protein concentrations in *Echinacea purpurea* (EP) (L.) Moench roots, crude extract was prepared from 50 g of raw EP roots’ material. Total protein was precipitated from crude extract by TCA-acetone and the quantity was estimated at 90.58 ± 1.74 mg by Bradford assay (Table 1).

Salting out with ammonium sulphate was performed in order to enrich the proteins with hemagglutinating activity. The 60% to 80% saturated ammonium sulphate fraction (F_60–80_) contained 11.4 ± 0.7 mg of protein and the remaining 100% saturated ammonium sulphate fraction contained only 1.3 ± 0.4 mg of protein.

Crude EP root extract demonstrated relatively high hemagglutinating activity expressed as a hemagglutinating titre 1:16. F_60–80_ showed higher hemagglutinating activity, with an observed hemagglutination titre of 1:32. Fraction F_60-80_ was used for further protein purification steps.

**Figure 1:**
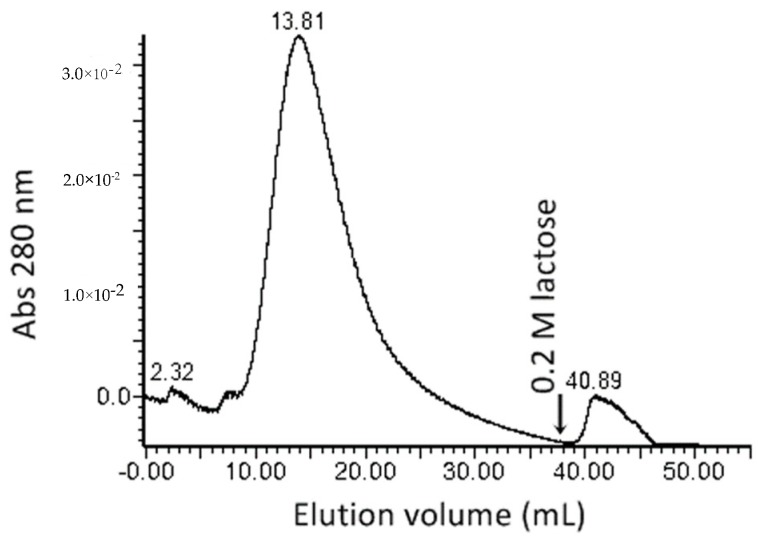
Lectin purification chromatogram. The 60–80% ammonium sulphate fraction was applied to the Sepharose-mannose column (1 × 11 cm), equilibrated with PBS buffer, pH 7.4. After the buffer change to 0.2 M lactose in PBS buffer, pH 7.4, at 40 mL, lectin was eluted. Fractions (2 mL) of proteins were collected and monitored for protein content at 280 nm.

Mannose specific binding was utilized for the affinity purification of proteins with hemagglutinating activity. Proteins with the hemagglutinating activity were purified from a F_60–80_ protein fraction using a Sepharose-mannose affinity chromatography. The addition of a 0.2 M lactose-containing buffer eluted 4.08 ± 0.8 μg of the hemagglutinating active protein in a single 5 mL (Figure 1, Table 1) fraction. The minimal protein concentration, demonstrating the hemagglutinating activity, was 1.02 μg mL^−1^.

### 2.2. Biochemical Characterisation of Purified Lectin

#### 2.2.1. Hemagglutinating Activity Inhibition by Carbohydrates Test Results

Lectins in our tested protein sample were strongly specific to lactose. Lactose solution, at a quantity of 3 mM, inhibited lectin-induced rabbit red blood cell agglutination (Table 2). Lectins demonstrated a bit less specificity to D-galactose and D-mannose molecules. However, D-glucose did not inhibit lectin activity. According to the literature, lactose specific lectins demonstrated similar specificity characteristics towards carbohydrates. For example, other studies on the haemagglutination activity of plant lectins have found that the abovementioned activity of lectin from *Cinachyrella apion* L. thallus was inhibited by lactose, however D-glucose and D-galactose did not show the same inhibiting effect [21]. Furthermore, D-mannose specific lectins were purified from *Helianthus annuus* L. roots, which are genetically related to purple coneflower [22].

Results of hemagglutination inhibition showed that the lectins in *Echinacea purpurea* L. (Moench) roots were specific to the D-glucose enantiomers, i.e., D-mannose, D-galactose, but not to D-glucose itself. We can assume that an epimeric D-galactose configuration and a disaccharide (lactose) molecular size are important to the tested lectin specificity. However, D-glucose’s stereoisomeric configuration in the lactose molecule is not important in its interaction with the lectin carbohydrate binding centre.

#### 2.2.2. Purified Hemagglutinating Active Fraction Analysis by SDS-PAGE and Western Blot

**Figure 2:**
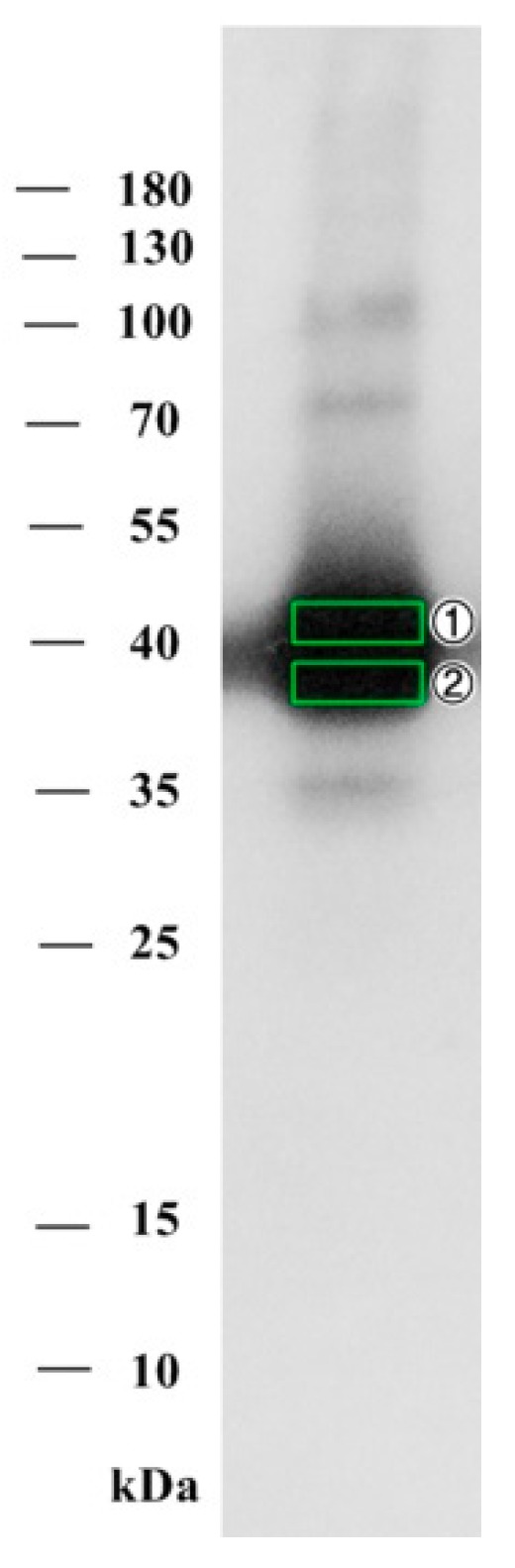
Glycoprotein visualization in hemagglutinating active fraction by immunoblotting with anti-xylose antibody. Marked areas No. 1 and No. 2 were excised and analyzed by mass spectrometry.

In order to visualize targeted proteins, immunochemical analysis methods can be used. Glycosylated proteins of plant origins can be detected with antibodies that are specific to plant cell glycosylation patterns and specific sugars, like xylose. The protein fraction, containing hemagglutinating activity, was analyzed by SDS-PAGE electrophoresis and immunoblotting using an anti-xylose antibody which was specific to glycosylated proteins. Several protein bands were identified. The most prominent was the ~40 kDa band with a diffused staining characteristic of glycosylated proteins. Another four bands at 100 kDa, ~72 kDa, ~55 kDa, and ~35 kDa were visible (Figure 2).

#### 2.2.3. Lectin Identification

**Figure A1:**
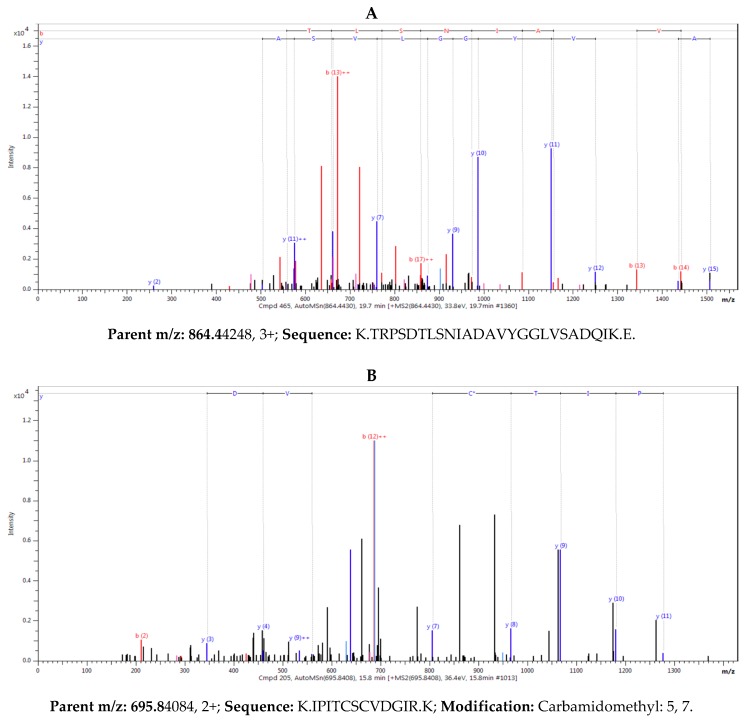
MS spectra (**A**,**B**) of the two identified peptides with match to the *Echinacea purpurea* trancriptome locus epa_locus_7790_iso_1_len_1277_ver_2 or *Helianthus annus* genome locus Ha412v1r1_08g009630.

*Echinacea’s* transcriptome-based mass fingerprinting of the purified glycoprotein fraction identified the epa_locus_7790_iso_1_len_1277_ver_2 (EP-7790), representing the 40.1 kDa protein with a peptidoglycan binding lysin domain (Table A1, Figure A1). Since the transcriptome database represents only the expressed genome fraction, the analysis was confirmed by using the genome database of a related plant *Helianthus annuus*. The identified locus Ha412v1r1_08g009630 corresponded to a glycoprotein homologous of the *Echinacea’s* locus EP-7790. The remaining two proteins were identified by the mass fingerprinting analysis and annotated as the glycerophosphodiester phosphodiesterase GDPDL3-like and early nodulin-like protein 2 isoform X4. The latter has not been reported to have haemagglutination activity and might represent impurities in the glycoprotein’s preparation.

**Figure A2:**
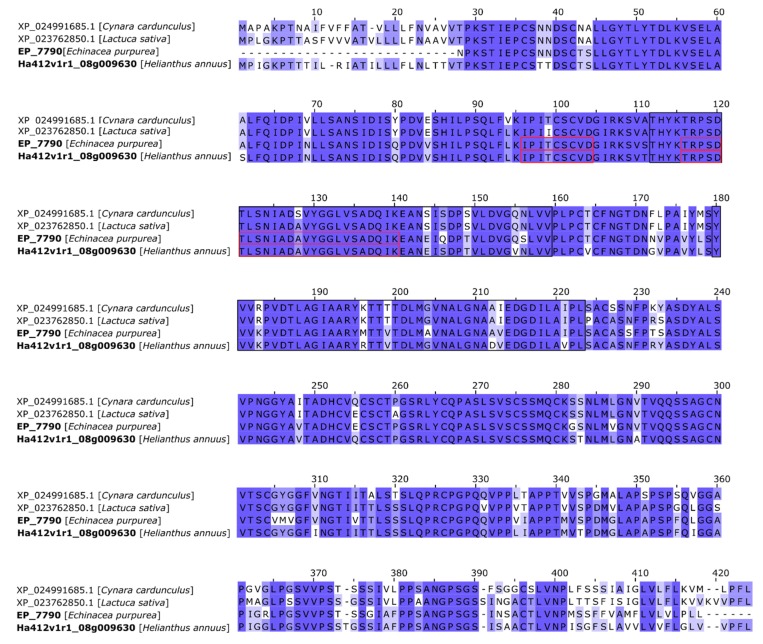
Multiple sequence alignment of identified LysM domain-containing proteins in *Echinacea purpurea* L. (Moench), *Helianthus annuus* L., *Lactuca sativa* L., and *Cynara cardunculus* L. Amino acid sequences coding LysM domain are boxed in black. ■ indicates positions which have a single, fully conserved residue; ■ indicates conservation between groups of strongly similar properties; ■ indicates conservation between groups of weakly similar properties. Sequence of peptides identified by mass fingerprint are indicated by red lines.

Results of the Blast search revealed that the *Echinacea* EP-7790 sequence was highly homologous (sequence identity was from 83% to 90%) to LysM domain-containing proteins in *Helianthus annuus* L., *Lactuca sativa* L., and *Cynara cardunculus var. scolymus* L (Table 3; Figure A2).

The sunflower’s (*Helianthus annuus* L.) LysM domain-containing protein anchored with glycosyl phosphate (XP_021976452.1) was identical to the Ha412v1r1_08g009630 sequence identified in *Helianthus annuus* L. genome’s database and demonstrated the highest identity (90%) to the protein encoded in locus EP-7790.

### 2.3. Purified LysM Lectin Impact to Kidney Morphology

**Figure 3:**
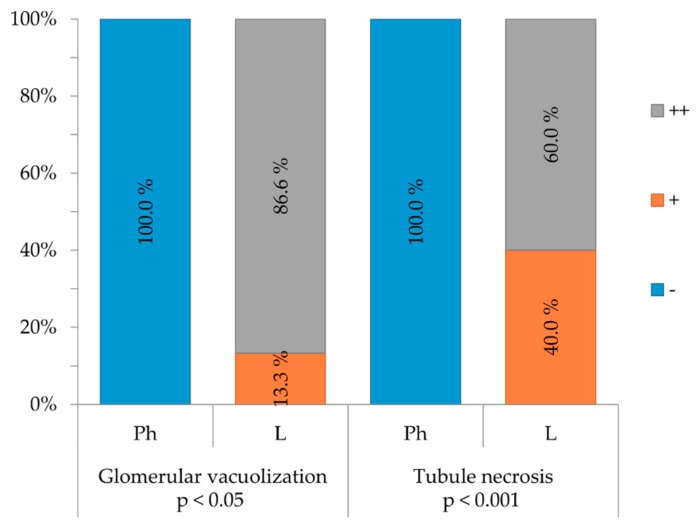
Morphological changes (glomerular vacuolization and tubule necrosis) in kidneys are represented. Occurrence (-) none, (+) mild, and (++) are marked, animal groups are abbreviated as Ph-injected with NaCl 0.9%, L-injected with *Echinacea purpurea* (L.) Moench (EP) root LysM lectin.

**Figure A3:**
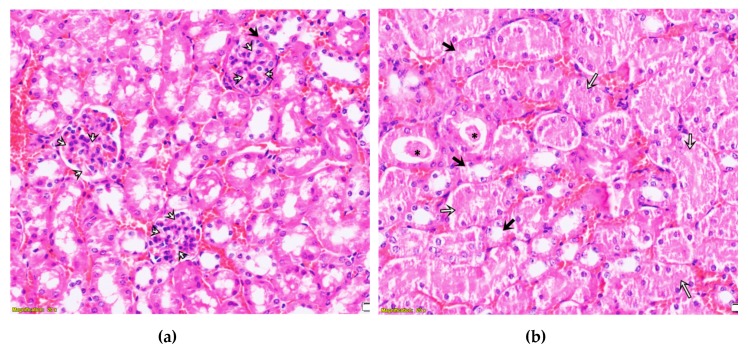
Histopathology of the kidney of BALB/c mice receiving LysM lectin fraction: (**a**) Glomerular morphology disruptions. White arrows show vacuolated cells in glomeruli, the black arrow indicates Bauman’s space filled with proteinaceous fluid infiltration; (**b**) tubular morphology disruption. The black asterisk shows tubular protein cylinders; black arrows indicate distal tubular necrosis, white arrows indicate proximal tubular necrosis.

**Figure A4:**
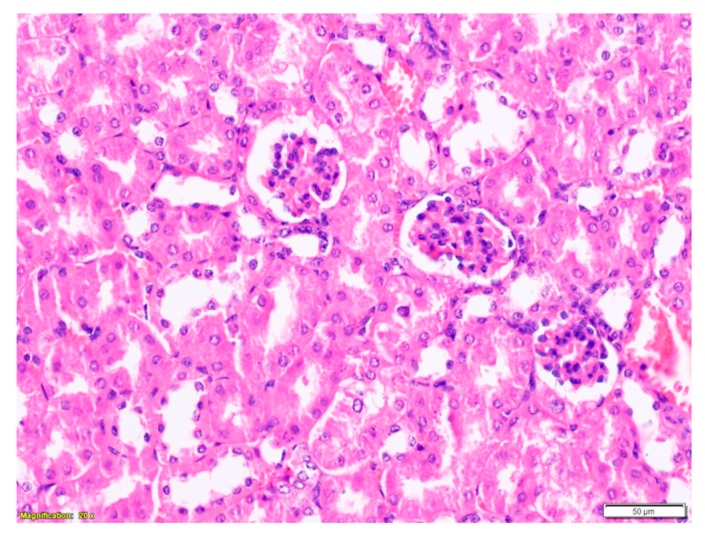
Histology of the kidney of BALB/c mice receiving NaCl 0.9% injections.

The kidney is one of the organs that carries out the function of metabolism and elimination of a drug. Glomeruli are the primary site for removal of macromolecules from the vascular circulation. Therefore kidney’s morphological and morphometric changes under the effect of lectins were evaluated. None of the investigated animal groups developed a visible kidney inflammation, however statistically significant (*p* < 0.001) tubule necrosis and glomerular vacuolization (*p* < 0.05) was observed (Figure 3). Appendix B (Figure A3 and Figure A4) demonstrates morphological changes observed in Balb/c mice treated with LysM lectin fraction. Glomerular vacuolization Appendix B (Figure A3a and Figure A4), necrotic tubular epithelium (pyknotic nuclei and acidophilic cytoplasm), and tubular protein cylinders in the kidney’s cortex and medulla Appendix B (Figure A3b and Figure A4) were observed. Protein cylinders deposit as a consequence of tubular damage, which indicates a disrupted tubular secretion.

**Figure 4:**
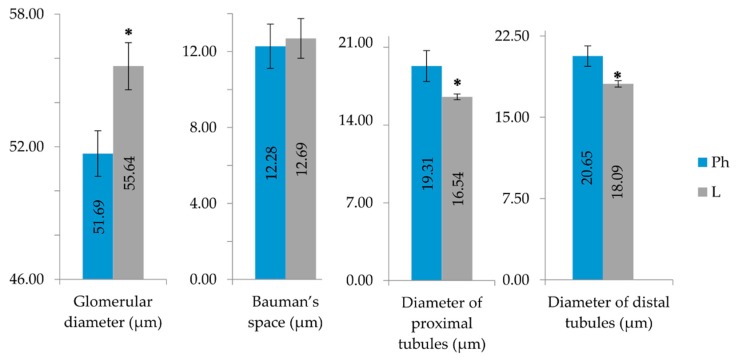
Comparison of morphometric changes in mice kidneys. Animal groups abbreviated as: Ph-injected with NaCl 0.9%, L-injected with EP root LysM lectin.

Statistically significant (*p* < 0.05) morphometric changes in the diameter of the kidneys’ nephrons’ proximal and distal tubules were observed (Figure 4). The statistically significant increase of glomerular diameter might be caused by increased cellular hypertrophy and hyperplasia as well as increased hyperfiltration. The statistically significant decrease of the inner diameter of distal and proximal tubules indicates swelling of tubular cells caused by cell structural disruptions.

## 3. Discussion

Hemagglutinating active glycoproteins of purple coneflower’s fresh root are ~40 kDa size and contain peptidoglycan binding LysM domains that are specific to lactose, D-mannose, and D-galactose. In 2008, E. Van Damme and colleagues [23] distinguished 12 plant lectin families according to their domains and carbohydrate specificity. In the same publication, LysM domains were considered as a lectin-like domain family. A decade later, A. C. Ribeiro and others (2018) [1] proposed the classification of lectins in families, based on structural and evolutionary relationships among their carbohydrate binding domains. These authors excluded the LysM domain’s lectin family. This domain can act as a separate lectin or as an active part in another lectin’s structure.

LysM domain-containing protein functions have been mainly studied in plant models such as *Medicago truncatula*, *Arabidopsis thaliana*, *Oryza sativa*, or *Lotus japonicus*. It is well established that LysM functions in plant defence and symbiosis with bacteria [24,25,26]. Phylogenetic analyses indicated that ancient LysM domains evolved through local and segmental duplications [27] and were widely spread in the kingdom *Plantae* and across other kingdoms too [28]. A structural analysis of LysM domains revealed a βααβ structure containing two α-helices positioned to one side of a two-stranded antiparallel β-sheet. Moreover, the same structural features have been observed in different species, i.e., *Medicago sativa* and *Escherichia coli* bacteria [29,30,31], enabled them to be called conservative. A structural and functional analysis demonstrated LysM domain thermostability, depending on two disulfide bonds. It was known that LysM domains bind to various types of peptidoglycan and chitin, recognizing the N-acetylglucosamine (GlcNAc) moiety [27]. Elucidated stoichiometry of (GlcNAc)_n_/LysM was 1:1; moreover, domains were critical for (GlcNAc)_5_ binding [32]. Experimental data showed the domain to have the least affinity to smaller (GlcNAc)_2_ and (GlcNAc)_3_ oligomers [33]. Our data support these findings. Larger disaccharide lactose molecules were able to inhibit hemmagglutinating activity at small concentrations (3 mM), meanwhile, larger concentrations (21–42 mM) of monosaccharide molecules; i.e., D-galactose, D-mannose, or GlcNAc, were required.

Reversible carbohydrate-binding LysM properties [34] enabled hemagglutinating activity in *Echinacea purpurea* (L.) protein fractions on rabbits’ erythrocytes. According to transcriptomic data, the *Echinacea purpurea* (L.) Moench plant might contain other lectins, such as jacalin-like lectin domain containing protein, lectin receptor kinases, G-type lectin S-receptor-like serine/threonine-protein kinase SD3-1, S locus lectin protein kinase family protein, with possible hemmagglutinating activities. Therefore, it might be that our identified LysM domain containing lectin is not the only one responsible for the observed nephrotoxic effects. A more detailed characterization of 40 kDa lectin is required.

Results of the experiment in vivo demonstrated the nephrotoxicity of purified *Echinacea purpurea* (L.) LysM lectin: Vacuolated cells in glomeruli, Bauman’s space filled with proteinaceous fluid infiltration, tubular protein cylinders, distal and proximal tubular necrosis. Due to carbohydrate’s specificity, plant lectins are able to bind to glomeruli, and distal and proximal tubules [35]. Previous studies showed that galactose/GalNAc specific lectins induced mitogen activated protein kinases (MAPK) [36] responsible for gene expression, mitosis, and metabolism to motility, survival and apoptosis, and differentiation [37]. Further data showed dysregulation of normal MAPK signaling was implicated in both acute and chronic kidney injuries [38]. Moreover, complexes of macromolecules, circulating through the glomeruli, may be deposited in subepithelial, subendothelial, or mesangial locations. The immune deposits are capable of triggering a sequence of inflammatory responses such as recruitment and localization of inflammatory cells at the site, release of inflammatory mediators and enzymes, destruction of glomerular structures [39]. This mechanism may damage the glomerular filtration barrier. However, various substances (such as lectins) may damage the filtration barrier themselves. Once the substance deposits in the glomeruli, it may cause an obstruction and reduced perfusion of the glomeruli. As a result, the glomerular filtration reduces and a smaller amount of primary urine (including sodium chloride) reaches the distal tubule where *macula densa* is situated. In response to decreased sodium chloride in the primary urine, the *macula densa* cells trigger juxtaglomerular cells to secrete more renin. Renin increases the blood pressure via the renin–angiotensin–aldosterone system. Glomerular hyperfiltration (hyperemia) is the result of increased hydrostatic pressure, which might damage glomerular capillaries and the glomerular filter, eventually causing a glomeruli leakage [40]. Glomerular hyperemia may also be caused by inflammatory mediators that cause the dilation of renal blood vessels [41]. Large quantity of substances might leak into the proximal tubule because of the impaired filter. Huge amounts of albumin may overload the protein reabsorption capabilities of the proximal convoluted tubular epithelium, causing protein-rich glomerular filtrate to accumulate in the variably dilated tubular lumina and, therefore, causing protein to appear in the urine [42]. Because of proteinuria, albumin which is reabsorbed by proximal tubular epithelial cells by receptor-mediated endocytosis accumulates in the tubular cells. Chemoattractants are released in response to continuous albumin exposure, and so tubulointerstitial inflammation may occur. This condition may eventually cause degeneration and necrosis of the tubules [43,44].

## 4. Materials and Methods

### 4.1. Herbal Material Preparation

Fresh roots (100 g) of 3-year-old *Echinacea purpurea* L. (Moench) specimens were taxonomically identified and kindly provided by Professor Ona Ragazinskiene from the Kaunas Botanical Garden of Vytautas Magnus University’s collection, plant specimen number XX-0-KAUN-1960-O0087, Kaunas, Lithuania (54°52’N 23°54’E, 84 m above sea level), in November 2017. Voucher specimen-EP 133511 was deposited from Kaunas Botanical Garden of Vytautas Magnus University’s herbarium. Water from the surface of the washed roots was absorbed with paper towels. Cleaned and drained roots were frozen in a freezer at −20 ± 2 °C and kept until the protein’s extraction.

### 4.2. Preparation of Crude Extract and Protein Precipitation

All procedures were carried out at 4 °C. A total of 50 g of frozen *E. purpurea* roots were homogenized with liquid nitrogen in the cooled IKA M20 universal mill (IKA-Werke GmbH & Co, Germany). Fine root powder was suspended in 250 mL of Buffer A (supplemented with 2% PVPP) and extracted by constant stirring for 2 h. The extract was centrifugated at 8500 g for 20 min and the supernatant was used for protein precipitation.

Proteins were precipitated from a crude extract by the ammonium sulphate salting-out method. A first protein fraction was obtained by adding ammonium sulphate to 60% saturation, followed by constant stirring for 2 h. Precipitated proteins were collected by centrifugation at 8500 g for 20 min. Supernatant was subjected to precipitation with 80% saturated ammonium sulphate by stirring for 16 h, and the precipitated proteins were collected by centrifugation, as described above. Furthermore, the ammonium sulphate was added to 100% saturation and the precipitated proteins were collected by centrifugation after stirring for 10 h.

The ammonium sulphate-precipitated protein pellets were resuspended in 5 mL of Buffer A, and subjected to extensive 16 h dialysis against Buffer A with the buffer changed every 4 h using dialysis membrane with 10,000 Da molecular weight cut-off.

The protein concentration was measured using the Bradford assay [45] and bovine serum albumin standards. Three replicates of the extraction were performed, and protein concentration was estimated as an average with standard deviation (M ± SD).

### 4.3. Lectin Separation by Affinity Chromatography

A quantity of 1 mL of 60–80% ammonium sulphate fraction was loaded on the affinity column (1 × 11 cm), filled with Sepharose-mannose matrix, and equilibrated with 20 mL Buffer A at the flow rate 1 mL min^-1^ by a Waters semipreparative chromatography system. One millilitre of the 80% saturated ammonium sulphate fraction was loaded on to the column. Unabsorbed proteins were eluted out of the column within 30 mL of elution with Buffer A at the flow rate of 1 mL min^−1^. After removing the unbound material, the lectins were desorbed with 20 mL of Buffer B (which contained 0.2 M lactose). Eluted proteins were collected into 2 mL fractions. Chromatographic separation was performed three times, and the protein concentrations of the obtained protein fraction replicates were estimated three times as well. The protein concentration was expressed in terms of arithmetric mean with standard deviation (M ± SD).

### 4.4. Biochemical Characterisation of Purified Lectin

#### 4.4.1. Hemagglutination Assay

Rabbit blood erythrocytes were used for the hemmaglutination assay. Blood samples were purchased by the Lithuanian University of Health Sciences, the Vivarium of the Veterinary academy. All procedures were approved by the local Animal Ethical Committee, license No. G2-16. (July 21, 2014). Blood samples were mixed with 4% trisodium citrate anticoagulant at a 4:1 ratio. The erythrocytes were separated by centrifugation at 800 g for 15 min at 4 °C. Precipitated red blood cells (RBC) were resuspended in PBS solution (pH 7.4) at a 1:14 ratio and washed four times by centrifugation. One volume of erythrocytes was treated with 7 volumes of trypsin in PBS solution (1 mg mL^−1^), pH 7.4, for 1 h at 37 °C. Trypsin was removed by centrifugation, as described above. The cells were washed four times in PBS, pH 7.4, and resuspended as 2% RBC suspension (3.5 × 10^8^ cells mL^−1^) [46].

Flat-shaped 96-well microtiter plates were used in the hemagglutinating activity (HA) assay. Each well of the plate received 25 μL of the PBS buffer, pH 7.4, followed by the addition of 25 μL of a protein sample and 25 μL of 2% of the RBC suspension. PBS buffer was used as a negative control. After incubation at the temperature of 22 °C for 2 h, the test results were checked under the light microscope Olympus CX21 (Olympus Corporation, Japan) at the magnification of ×10. The hemagglutination assay was modified according to [47]. Each well in a flat-transparent bottom microtiter plate was filled with 25 µL PBS, pH 7.4. 25 µL of protein extract was pipetted to the first well and serially diluted. The dilution was repeated in 1 to 8 wells. Then, 25 µL of sample from the 8th well was eliminated. A quantity of 25 µl of 2% RBC suspension was added to each well and gently swirled for 10 s. Hemagglutination titre was assessed by light microscopy observation after 60 min incubation at a temperature of 20 ± 2 °C. Hemagglutination activity was defined as a titer reciprocal of the maximum dilution giving positive hemagglutination.

#### 4.4.2. Hemagglutination Inhibition Assay

The carbohydrate specificity of the lectin was determined in the microtiter plates described by Silva M.C.C. [48]. All carbohydrate inhibitors were dissolved in PBS at an initial concentration of 400 mM to be tested. Two-fold carbohydrate serial dilutions were made in a microplate and the final concentration in the last well was 0.4 mM. The following mono- and disaccharides were used: Xylose, ribose, fructose, D-glucose, D-galactose, D-mannose, lactose, sucrose, maltose, acetylgalactosamine, N-acetylglucosamine. A quantity of 50 µL (2 µg) of the lectin solution was added to the two-fold serially diluted solutions of carbohydrate-inhibitor, at a final volume of 100 µL, and the plate was incubated for 20 min at a temperature of 20 ± 2 °C. Then, 50 µL of rabbit erythrocytes (2% v/v suspension) was added to each well and allowed to incubate for 60 min at a temperature of 20 ± 2°C. The degree of haemagglutination was examined and the maximum dilution of the inhibitor solution showing the inhibition was recorded. The controls were set up with saline and erythrocytes, sugar and erythrocytes, and lectin and erythrocytes. Results were expressed as the minimal sugar or glycoprotein concentration required to inhibit haemagglutinating doses of the lectin.

#### 4.4.3. SDS-PAGE and Western Blotting

Active protein fraction was analyzed by SDS-PAGE on 12% gel slab with 5% stacking gel by Lämmli [49] using a Protean Mini electrophoresis unit (Bio-Rad, Hercules, CA, USA). PageRuler (Thermo-Fisher Scientific, Vilnius, Lithuania) molecular marker was used as protein size reference. Proteins were transferred to polyvinylidene fluoride (PVDF) membrane using Fastblot semi-dry electrophoretic transfer apparatus (Biometra, Göttingen, Germany) for 70 min at 90 mA per stack.

The membrane was washed three times for 10 min in TBS buffer, pH 7.4, blocked in blocking buffer for 1 h, and incubated with a primary rabbit anti-xylose (Dako, Glostrup, Denmark) antibody at 1:2500 dilution on rotating platform overnight at 4 °C. The membrane was washed with TBS-T and incubated in a secondary anti-rabbit antibody used at dilution 1:10 000 on a rotating platform for 1 h at 22 °C. After three 10 min washes in TBS-T buffer, the membrane was washed with an alkaline phosphate buffer for 5 min. The antibody labelling was detected by enhanced chemiluminescence using CDP Star solution (Sigma-Aldrich, Steinheim, Germany) and visualized using X-ray film (Kodak, Morstel, Belgium).

#### 4.4.4. Protein In-Gel Digestion, Sample Preparation, and Mass Spectrometry Analysis

Protein bands were excised from protein gels and digested with trypsin, with reference to [50]. Sliced gel slabs were incubated in destaining solution containing 25 mM NH_4_HCO_3_, 50% acetonitrile (ACN) for 10 min. Destained gel slabs were soaked in ACN and dehydrated for 10 min. The liquid was fully removed and 50 µL of trypsin solution (13 ng µL^−1^ trypsin, 5 mM NH_4_HCO_3_, 10% ACN) was added to the dry gel pieces and incubated at 37 °C for 16 h. Digested peptides were extracted with 200 µL of water for 10 min, followed by extraction with 200 µL of 50% acetonitrile. Both extraction liquids were combined and dried in vacuum centrifuge.

Peptides were desalted according to [51,52]. Samples were reconstituted in 60 µL of wash solvent (2% ACN, 0.1% trifluoroacetic acid), vortexed for 45 s, and centrifuged at 3000 g for 1 min. Sixty microliters of wetting solvent (80% ACN, 0.1% trifluoroacetic acid) was pipetted onto the Stage Tip/Tube assembly and centrifuged at 450 g for 2 min followed by 60 µL of washing solvent and centrifuged, as mentioned above. Samples were pipetted into the stage tip and centrifuged. The tip was washed twice with wash solvent by centrifugation. Cap/stage tip assembly was placed on a new eppendorf tube and peptides were eluted with the elution solvent (60% ACN, 0.1% trifluoroacetic acid) by centrifugation. Desalted peptide mixture was dried in a vacuum centrifuge and reconstituted in the 2% ACN, 0.05 % formic acid solvent.

Peptides were analyzed by a liquid chromatography-tandem mass spectrometry (LC-MS/MS), equipped with Ultimate3000 RSLC (Thermo-Scientific, Pitsburg, PA, USA) and Maxis 4G with Captive Spray ionisation source (Bruker-Daltonics, USA). Protein identification was carried out using Mascot software (Matrix Science, USA), using *Helianthus annuus* L.genome HA412v.1.0 [53] and *Echinacea purpurea* L. (Moench) transcriptome (http://medicinalplantgenomics.msu.edu/pub/data/MPGR/) databases, with the criteria; Mascot score ≥50, peptide number ≥2.

A BLAST P (default parameters) [54,55] search was performed to protein sequences identified in *E. purpurea* L. (Moench) transcriptome, in order to evaluate protein sequence identity and similarities in other plants. To evaluate the similarity between the identified homologous protein sequences, the multiple sequence alignment was performed using Clustal Omega software [56].

### 4.5. Evaluation of LysM Lectin Biological Activity In Vivo

#### 4.5.1. Animals

Male Balb/C mice (8 weeks old, 18–20 g, n = 30) were acquired from Lithuanian University of Health Sciences, Academy of Veterinary, Vivarium. Animals were kept under the standard conditions in the Biological Research Center under the optimized hygienic conditions with 12:12 h light-dark cycle. They were fed with a standard pellet diet and water ad libitum. Experiments were approved by State Food and Veterinary Service, February 7, 2017 permission No. G. 2-56.

#### 4.5.2. Experimental Model

Two different test animal groups were selected: Animals (n = 15) in the negative control group got 50 μL peritoneal injections of physiological NaCl solution; animals in the lectin group (n = 15) got a 50 μL (250 μg/kg) dose of purified LysM lectin peritoneal injection. Injections were repeated 4 times every 7 days. On the 5th week, animals were sedated with carbon dioxide and euthanized by cervical dislocation. Both kidney samples from each animal were aseptically removed, fixed in 10% neutrally buffered formalin, and paraffin embedded.

#### 4.5.3. Histological Analysis of Kidney Samples

Kidney samples were cut at 3 μm thickness and stained with hematoxylin and eosin (H&E) for a light microscopic examination. The analysis was performed using the Olympus light microscope fitted with a digital camera Olympus DP72 and using the CellSensDimension and Image-Pro Plus software. Morphological examination included observation of an inflammation, necrosis, medullary and cortex ischemia, glomerular necrosis and hyperemia, tubular necrosis. A score was assigned to each parameter as follows: (0) None, (1) mild, (2) marked. A morphometric analysis was performed: Glomerular diameter, Bauman’s space, and proximal and distal tubules’ inner diameters were measured. The maximum profile area of glomeruli was measured using glomeruli sectioned through and perpendicular to the hilum and/or capsular origin of the proximal tubule. To correct for slight differences in apparent glomerular size, based on the thickness of tissue sections, a maximal profile area of 20 glomeruli was measured in five different sections, four glomeruli on each section, for each case. Glomeruli that were badly distorted or tangentially sectioned were excluded from analysis [50]. Images of the histological sections were captured using 20× objective lense and were analyzed with Image-Pro Plus software. A double-blinded sample analysis was performed by two independent pathologists.

### 4.6. Statistical Analysis

The data were expressed as a mean ± SD. A probability *p* < 0.05 was considered to be significant. Statistical comparison between the groups was performed using two-way analysis of variance (ANOVA) with the Wilkinson–Mann–Whitney test, nested ANOVA with the Levene test, and post hoc analysis with Tuckey‘s test. SPSS ver. 22.0 was used for calculations.

## Figures and Tables

**Table 1:** Quantity and hemagglutinating activity of purified proteins from 50 g of *Echinacea purpurea* (L.) Moench roots.

	Total Protein (mg)	Specific Activity ^a^ (HU, mg Protein^−1^)	Amount of Lectin ^b^ (μg mL^−1^)	Purification Fold ^c^
Crude extract	90.58 ± 1.74	5.66	113.22	1
F_60-80_	11.38 ± 0.71	0.35	35.56	3.18
Purified LysM lectin	0.04 ± 0.008	0.00204	1.02	111.00

^a^ Specific activity (HU) shown as the ratio between hemagglutinating activity and protein content; ^b^ minimal concentration of protein able to cause visible agglutination of 2% suspension of rabbit erythrocytes; ^c^ purification index was calculated as the ratio between the minimal concentration of crude extract able to cause visible agglutination of rabbit erythrocytes and that of the protein fraction obtained at each purification step.

**Table 2:** Minimal inhibitory concentration (MIC) of carbohydrates specific to purple coneflower fresh root lectins. NI: No inhibition detected.

	Minimum Inhibitory Concentration (mM)
Lactose	3
D-mannose	21
D-galactose	21
Fructose	42
Sucrose	42
Maltose	42
N-Acetylglucosamine	42
Ribose	NI
D-glucose	NI
D-Xylose	NI
Acetyl-galactosamine	NI

**Table 3:** *Echinacea* transcriptome observed glycoproteins‘ identity with proteins from other plant species.

	Description	Max Score	Total Score	Query Coverage	E value	Percent Identity	Accession Number in *NCBI Protein* Database
*epa_locus_7790_iso_1_len_1277*	LysM domain-containing GPI-anchored protein [*Helianthus annuus*]	676	676	96%	0.0	90	XP_021976452.1
	LysM domain-containing GPI-anchored protein [*Lactuca sativa*]	629	629	97%	0.0	85	XP_023762850.1
	LysM domain-containing GPI-anchored protein [*Cynara cardunculus var. scolymus*]	646	646	99%	0.0	83	XP_024991685.1

## Appendix A

**Table A1:** Results of protein identification using LC-MS/MS and *Echinacea purpurea* trancriptome or *Helianthus annus* genome database and sequence annotation using NCBI Blast.

							NCBI Blast Annotation	
Database Accession	MW, kDa	pI	Mascot Score	Number of Peptides	SC, %	RMS90, ppm	NCBI RefSeq Accession	Protein Name
*Echinacea purpurea* transcriptome								
epa_locus_7790_iso_1_len_1277_ver_2	40.1	4.5	184.6	2	9.5	6.21	XP_023762850.1	lysM domain-containing GPI-anchored protein 1-like
epa_locus_17753_iso_4_len_2582_ver_2	82.3	5	431.0	6	8.8	5.51	XP_022020643.1	glycerophosphodiester phosphodiesterase GDPDL3-like
*Helianthus annus* genome								
Ha412v1r1_08g009630	43.3	4.7	182.0	2	8.8	6.21	XP_021976452.1	lysM domain-containing GPI-anchored protein 1-like
Ha412v1r1_11g016880	37.6	6.3	259.2	2	5.4	4.09	XP_021992913.1	early nodulin-like protein 2 isoform X4
